# Sex-Specific Trends in Thyroid Cancer Incidence and Histological Patterns in Northern Tunisia: A Population-Based Study with Implications for Cancer Control and Prevention

**DOI:** 10.3390/cancers18091472

**Published:** 2026-05-03

**Authors:** Hyem Khiari, Soumaya Henchiri, Ismail Dergaa, Halil İbrahim Ceylan, Valentina Stefanica, Saida Sakhri, Semia Zarraa, Hajer Ben Mansour, Yoser Zenzri, Houssem Dziri, Nadia Ben Mansour, Najet Mahjoub, Raul Ioan Muntean, Mohamed Hsairi

**Affiliations:** 1 Department of Epidemiology, Salah Azaiez Institute of Cancer, Bab Saadoun, Tunis 1006, Tunisia; houyem.khiari@fmt.utm.tn (H.K.); houssem.dziri@etudiant-fmt.utm.tn (H.D.); mohamed.hsairi@yahoo.fr (M.H.); 2 Directorate of Basic Healthcare, Tunis 1002, Tunisia; drhenchiri.soumaya@yahoo.com; 3 High Institute of Sport and Physical Education of Ksar Said, University of Manouba, Manouba 2010, Tunisia; phd.dergaa@gmail.com; 4 Physical Activity Research Unit, Sport and Health (UR18JS01), National Observatory of Sports, Tunis 1003, Tunisia; 5 High Institute of Sport and Physical Education of Kef, University of Jendouba, Jendouba 7100, Tunisia; 6 Physical Education and Sports Teaching Department, Faculty of Sports Sciences, Atatürk University, Erzurum 25240, Türkiye; 7 Department of Physical Education and Sport, Faculty of Sciences, Physical Education and Informatics, Pitesti University Center, National University of Science and Technology Politehnica Bucharest, 060042 Pitesti, Romania; valentina.stefanica@upb.ro; 8 Department of Surgical Oncology, Salah Azaiez Institute of Cancer, Tunis 1007, Tunisia; saida.sakhri@fmt.utm.tn; 9 Department of Radiotherapy, Salah Azaiez Institute of Cancer, Tunis 1007, Tunisia; semia.zarraa@yahoo.com; 10 Department of Medical Oncology, Salah Azaiez Institute of Cancer, Tunis 1007, Tunisia; hajer.benmansour@fmt.utm.tn (H.B.M.); yosr.zenzri@fmt.utm.tn (Y.Z.); 11 Charles Nicolle Hospital, Tunis 1006, Tunisia; nadia.benmansour@fmt.utm.tn; 12 Department of Medical Oncology, Jendouba Hospital, Jendouba 8100, Tunisia; mahjoubnajet@yahoo.fr; 13 Department of Physical Education and Sport, Faculty of Law and Social Sciences, University “1 Decembrie 1918” of Alba Iulia, 510009 Alba Iulia, Romania

**Keywords:** thyroid cancer, cancer epidemiology, population-based registry, incidence trends, joinpoint regression, overdiagnosis, cancer prevention, sex differences, papillary thyroid carcinoma, Tunisia

## Abstract

Background: Thyroid cancer (TC) represents the most common endocrine malignancy worldwide, with incidence increasing rapidly across diverse geographic regions. However, population-based evidence from North Africa remains limited, and comprehensive longitudinal analyses examining sex-specific incidence patterns, histological subtypes, and trends in tumor extension are lacking in Tunisia. Aim: This study aimed to (i) quantify TC incidence trends by sex and age group, (ii) characterize histological subtype-specific temporal patterns and tumor extension at diagnosis in northern Tunisia between 2000 and 2018, and (iii) to address projections in incidence by sex until 2040. Methods: A retrospective, population-based registry study was conducted using data from the Northern Tunisia Cancer Registry (NTCR), covering 11 governorates with a population of 5,233,700 in 2018. All primary invasive TC cases diagnosed between 2000 and 2018 were included (n = 3639). Age-standardized incidence rates (ASIRs) were calculated using the WHO standard population. Temporal trends were assessed using Joinpoint regression to estimate average annual percentage change (AAPC) with 95% confidence intervals. Projections of TC incidence to 2040 were generated using Bayesian autoregressive age–period–cohort models. Results: TC incidence increased significantly between 2000 and 2018, with overall ASIR rising from 2.8 to 5.0 per 100,000 person-years (AAPC = 3.8%, *p* < 0.001). In males, ASIR increased from 0.9 to 2.4 (AAPC = 3.0%, *p* < 0.001), while in females it rose from 3.7 to 7.8 (AAPC = 4.3%, 95% CI: 3.0–5.7; *p* < 0.001). The increase was predominantly driven by papillary thyroid carcinoma (PTC) (AAPC = 6.4% in males; 5.8% in females; both *p* < 0.001), whereas follicular thyroid carcinoma (FTC) remained stable. Notably, the proportion of metastatic cases decreased significantly in females (AAPC = −7.2%, *p* = 0.033), and the proportion of regionally advanced disease decreased in males (AAPC = −5.0%, *p* = 0.034). Conclusions: This population-based study demonstrates a sustained rise in TC incidence in northern Tunisia, disproportionately affecting women and largely driven by papillary histology. The concurrent increase in TC incidence alongside a reduction in regional and metastatic extension at diagnosis occurred. These findings have important implications for cancer prevention and control, highlighting the need for risk-adapted screening strategies and rationalized diagnostic practices.

## Simple Summary

Thyroid cancer is one of the fastest-increasing cancers worldwide, but it remains unclear whether this rise reflects a true increase in disease or improved detection of small, low-risk tumors that may never cause harm. In Tunisia, most previous studies have been limited to single hospitals and short time periods, making it difficult to understand long-term population-level trends. In this study, we analyzed nearly two decades of population-based cancer registry data from northern Tunisia to examine how thyroid cancer rates have changed over time, including differences by sex, age group, tumor type, and disease stage at diagnosis. Our findings provide the first comprehensive overview of thyroid cancer trends in this region and help clarify whether rising diagnoses reflect a true increase in disease burden or overdiagnosis, thereby supporting more effective prevention, screening, and clinical decision-making strategies.

## 1. Introduction

Thyroid cancer occupies a singular position in global oncology: it is simultaneously one of the most rapidly rising malignancies and one of the least lethal, generating a persistent and clinically consequential discrepancy between incident case burden and disease mortality. According to GLOBOCAN 2022, approximately 821,000 new TC cases were diagnosed worldwide, positioning it as the seventh most common cancer overall, fifth among women, and thirteenth among men [1,2]. TC-related deaths numbered only 47,507 globally in 2022, ranking it twenty-fourth among causes of cancer mortality, a gap that reflects not only favorable tumor biology but also the profound influence of diagnostic intensity on case ascertainment [2]. The geographic distribution of incidence is strikingly heterogeneous. The highest rates are recorded in China (24.6/100,000), Turkey (15.6/100,000), and the United States (12.1/100,000), while Central and East Africa report rates below 1/100,000 [3]. Mortality follows a contrasting pattern: high-income countries maintain rates below 0.3/100,000, whereas North and Central Africa and the Middle East carry burdens ranging from 0.7 to 2.7/100,000 [3]. Tunisia occupies an intermediate position, with GLOBOCAN estimating an age-standardized incidence of 2.5/100,000 and a mortality rate of 0.44/100,000 [3]. The public health stakes extend beyond current figures: projections based on the Global Burden of Disease Study indicate that TC will rank as the fourth most common malignancy among adults aged 20 to 49 by 2040 [4], a trajectory with substantial implications for health systems operating under resource constraints. Generating reliable, disaggregated incidence data from population-based registries is therefore not merely a surveillance exercise but a prerequisite for any rational prevention and screening policy [5,6].

TC encompasses a histologically heterogeneous group of malignancies. Differentiated tumors, papillary TC (PTC) and follicular TC (FTC), account for over 90% of all diagnoses, with PTC representing 80–85% of cases in most registries worldwide [7,8]. These two subtypes differ substantially in their epidemiological dynamics. PTC incidence has risen sharply across high- and middle-income countries over the past three decades, a trend widely attributed to the widespread adoption of high-resolution neck ultrasonography and image-guided fine-needle aspiration biopsy, which detect small subclinical lesions that would otherwise remain clinically silent [9,10]. FTC, diagnosed primarily by histopathological assessment of capsular and vascular invasion, has not contributed to this rise and has remained stable in most settings [7]. The overdiagnosis hypothesis, that a substantial proportion of newly registered TC cases represent clinically inconsequential tumors detected solely through enhanced surveillance, is supported by a consistent epidemiological signature: rising PTC incidence coupled with stable or declining mortality and decreasing proportions of regional and metastatic extension of TC cases at diagnosis [8,9]. This pattern has been documented across diverse country settings, from South Korea and the United States to India and several European nations [11,12,13]. Sex is a fundamental modifier of TC risk. Women account for approximately three-quarters of all TC diagnoses globally [1,7], a disparity maintained across virtually every world region and across the full range of histological subtypes. Modifiable metabolic risk factors contribute an independent layer of risk. High body mass index (BMI) has been linked to both elevated TC incidence and more aggressive tumor biology [14,15], and a multi-country analysis spanning 204 nations identified tobacco use, second-hand smoke exposure, BMI, and physical inactivity as significant positive correlates of TC burden [5]. In Tunisia, the prevalence of obesity among women reached 34.6% by 2016, nearly double the male rate of 17.6%, and overall adult obesity increased from 27.2% in 2005 to 34.1% in 2016 [16]. These nationally documented trends provide a plausible metabolic substrate for the rising incidence of TC, particularly in women.

Despite these global developments, the epidemiological literature on TC in Tunisia consists primarily of single-center clinical series and conference abstracts rather than population-based longitudinal analyses [17,18]. This gap is consequential. Without robust registry-derived data stratified by sex, age, and histological subtype over multiple years, it is not possible to determine whether rising case counts in Tunisia reflect a genuine increase in disease burden, overdiagnosis, or a combination of both. This distinction is not academic. In South Korea, national recognition of overdiagnosis led to revised screening recommendations followed by a measurable decline in TC incidence [11]. In the United States, guideline changes issued by the American Thyroid Association (ATA) and the US Preventive Services Task Force between 2009 and 2017, discouraging biopsy of small, non-suspicious thyroid nodules and recommending active surveillance for micro-PTCs, preceded a downward trend in incidence [12,13]. The mechanisms generating rising incidence in low- and middle-income countries are less well characterized, with overdiagnosis and true risk factor increases likely operating concurrently [19]. Northern Tunisia, which covers approximately 49% of the national population across 11 governorates and has maintained a cancer registry operating under IARC standards since 1998, is uniquely positioned to generate the longitudinal, population-based evidence needed to address these questions at the national level. No prior study has examined long-term trends in TC incidence from this registry with the degree of sex, age, histological, and extension specificity required to inform policy.

Based on these identified gaps, the present study aimed to analyze trends in TC incidence in northern Tunisia over 19 years of systematic, registry-based surveillance. Specifically, the study aimed to (i) quantify TC incidence trends by sex and age group using Joinpoint regression and APC estimation, (ii) characterize histological subtype-specific trends for PTC and FTC across the study period, and (iii) examine temporal changes in tumor extension patterns by sex. Collectively, these analyses provide the first comprehensive, population-based epidemiological profile of TC in northern Tunisia and establish an evidence baseline for national prevention and screening policy development.

## 2. Materials and Methods

### 2.1. Ethical Approval

This study was conducted in accordance with the Declaration of Helsinki. The research protocol was reviewed and approved by the Ethics Committee of Jendouba Hospital, Tunisia. Data collection and management complied with Decree No. 2008-846 of 24 March 2008, governing the confidentiality of cancer registries in Tunisia. The NTCR also obtained authorization from the National Authority for Personal Data Protection on 27 April 2017. Given the retrospective registry design, individual informed consent was waived; all patient identifiers were anonymized prior to analysis.

### 2.2. Registry Structure and Data Sources

**Figure 1:**
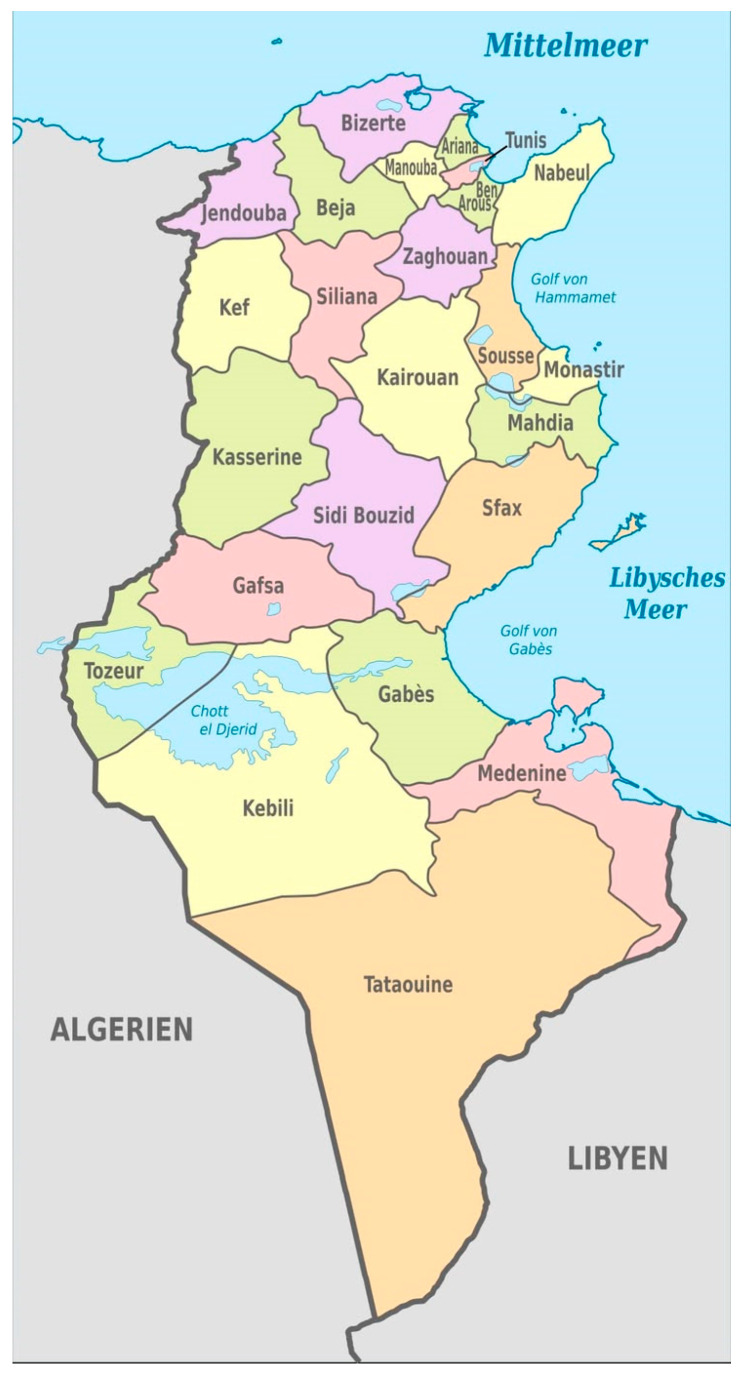
Map of Tunisia by governorate.

The Northern Tunisia Cancer Registry (NTCR) was established in 1998 as one of three regional, population-based cancer registries constituting Tunisia’s national cancer surveillance infrastructure. The NTCR covers 11 governorates across 28,162 km^2^, representing approximately one-fifth of the national territory (Figure 1). In 2018, this region hosted 5,233,700 inhabitants (2,636,160 males; 2,597,540 females), corresponding to 49% of the national population [20]. Population denominators by sex, age group, and year were obtained from Tunisia’s National Institute of Statistics for the period 2000 to 2018 [20].

The NTCR employs an active data collection method. Physician-registrars trained in accordance with IARC recommendations systematically review all cancer-related care sources across the public and private sectors in northern Tunisia, extracting data from medical and histopathological records [21,22]. Data entry was performed using EpiData software version 2.1b and stored in REC and CSV formats. The Department of Epidemiology at the Salah Azaiez Institute is responsible for data verification, coding, duplicate detection, and error correction. Tumor coding followed the International Classification of Diseases for Oncology (ICD-O), with TC coded as C73 [23].

Duplicate detection used a matching algorithm comparing name, sex, date of birth, address, year of incidence, social security number, histology number, and tumor characteristics [24,25]. Cross-referencing across public hospital records and private histopathology reports reduced residual duplication [26]. Regular quality training for registrars minimized systematic collection errors [27]. Systematic checks for sex/topography, age/topography, topography/histology, and age/topography/histology incompatibilities were conducted according to IARC standards [21,23].

### 2.3. Study Population

All primary invasive TC cases registered in the NTCR from 1 January 2000 to 31 December 2018 were eligible for inclusion. Recurrences, metastases, and cases with incomplete or unusable medical records were excluded. Variables extracted included patient sex, age at diagnosis, geographical origin, histological type (PTC, FTC, other), tumor extension (localized, regional, metastatic), and diagnostic basis.

### 2.4. Statistical Analysis

Data were analyzed using Excel (Microsoft Corporation, Redmond, WA, USA) and SPSS version 26.0 (IBM Corporation, Armonk, NY, USA). Categorical variables were expressed as frequencies and percentages, whereas quantitative variables were expressed as mean ± standard deviation (SD). Normality was assessed using the Kolmogorov–Smirnov test.

Incidence rates were calculated as the number of new TC cases per 100,000 person-years. Crude incidence rates (CIRs) and age-standardized incidence rates (ASIRs) were computed using the direct standardization method, with the WHO world standard population as the reference [21,28]. ASIRs permit valid comparisons across populations and over time by eliminating the confounding effect of differing age structures.

Trend analysis of age-standardized incidence rates (ASIRs) by age group and proportional distribution over the period 2000–2018 was performed using the Joinpoint Regression Program (version 4.9; National Cancer Institute, Bethesda, MD, USA) [29]. This method identifies statistically significant changes in temporal trends by fitting the simplest possible model to the observed data. Annual Percentage Changes (APCs) with 95% confidence intervals (CIs) were estimated for each segment, and the Average Annual Percent Change (AAPC) was calculated to summarize the overall trend across the entire study period. A log-linear regression model assuming Poisson variance was applied. The number of joinpoints was allowed to vary from 0 to 3, which is appropriate for a 19-year observation period. Model selection was based on the Weighted Bayesian Information Criterion (WBIC). A two-sided *p*-value < 0.05 was considered statistically significant.

Future thyroid cancer incidence rates were projected through 2040 using Bayesian autoregressive age–period–cohort (BAPC) models [30]. This approach was selected because it avoids unrealistic linear extrapolations inherent in classical APC models by allowing trends to evolve flexibly over time through a second-order random walk. The model was fitted to observed data from 2000 to 2018 using integrated nested Laplace approximations (INLA) for parameter estimation. Case counts were assumed to follow a Poisson distribution. Projections were stratified by sex to account for sex-specific demographic trends in thyroid cancer. All analyses were performed in R, and results are presented as age-standardized rates per 100,000 person-years with corresponding 95% confidence intervals (CIs).

## 3. Results

### 3.1. General Characteristics of Thyroid Cancer Cases

Between 2000 and 2018, the NTCR recorded 3639 primary invasive TC cases, averaging 202 per year. Women accounted for 78.5% of all cases, with a male-to-female sex ratio of 0.3. The mean age at diagnosis was 46.9 ± 16.3 years overall; males were diagnosed at a mean age of 50.9 ± 17.7 years compared with 45.9 ± 15.7 years in females. Table 1 presents case distribution by sex across the study period.

### 3.2. Overall and Sex-Specific Incidence Trends Results

**Figure 2:**
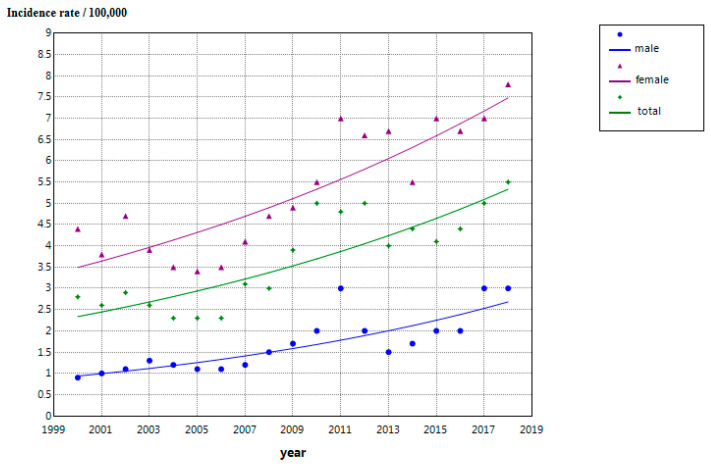
Trends in thyroid cancer incidence by gender in northern Tunisia, 2000–2018.

The overall ASIR increased from 2.8 per 100,000 person-years in 2000 to 5.0 per 100,000 in 2018, with an AAPC of 3.8% (*p* < 0.001). In males, the ASIR rose from 0.9 per 100,000 in 2000 to 2.4 per 100,000 in 2018 (AAPC = 3.0%, *p* < 0.001). In females, the ASIR increased from 3.7 per 100,000 in 2000 to 7.8 per 100,000 in 2018 (AAPC = 4.3%; 95% CI: 3.0–5.7; *p* < 0.001). Table 2 presents complete sex-stratified trend data, including ASIRs and AAPC estimates. Figure 2 displays the temporal trajectory of ASIRs by sex from 2000 to 2018.

### 3.3. Age-Specific Incidence Trends by Sex

**Figure 3:**
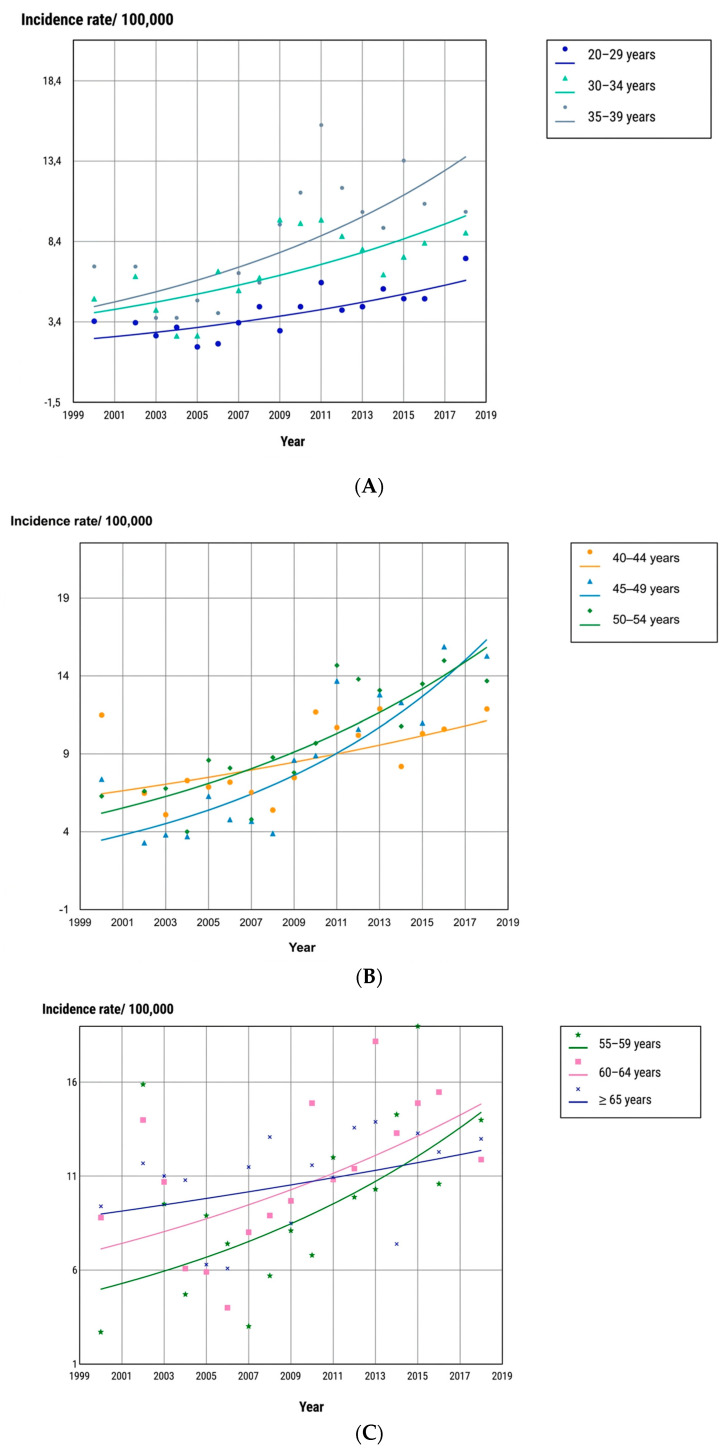
Trend in thyroid cancer incidence by age group in females (**A**–**C**) in northern Tunisia over the period 2000–2018.

Among women, TC incidence increased significantly across most age groups. Statistically significant upward trends were observed between 30 and 65 years of age. Only those aged 65 years or older showed stable trends across the study period. Table 3 presents age-group-specific incidence rates and AAPC estimates for females. Figure 3 displays the age-specific incidence trajectories among women.

**Figure 4:**
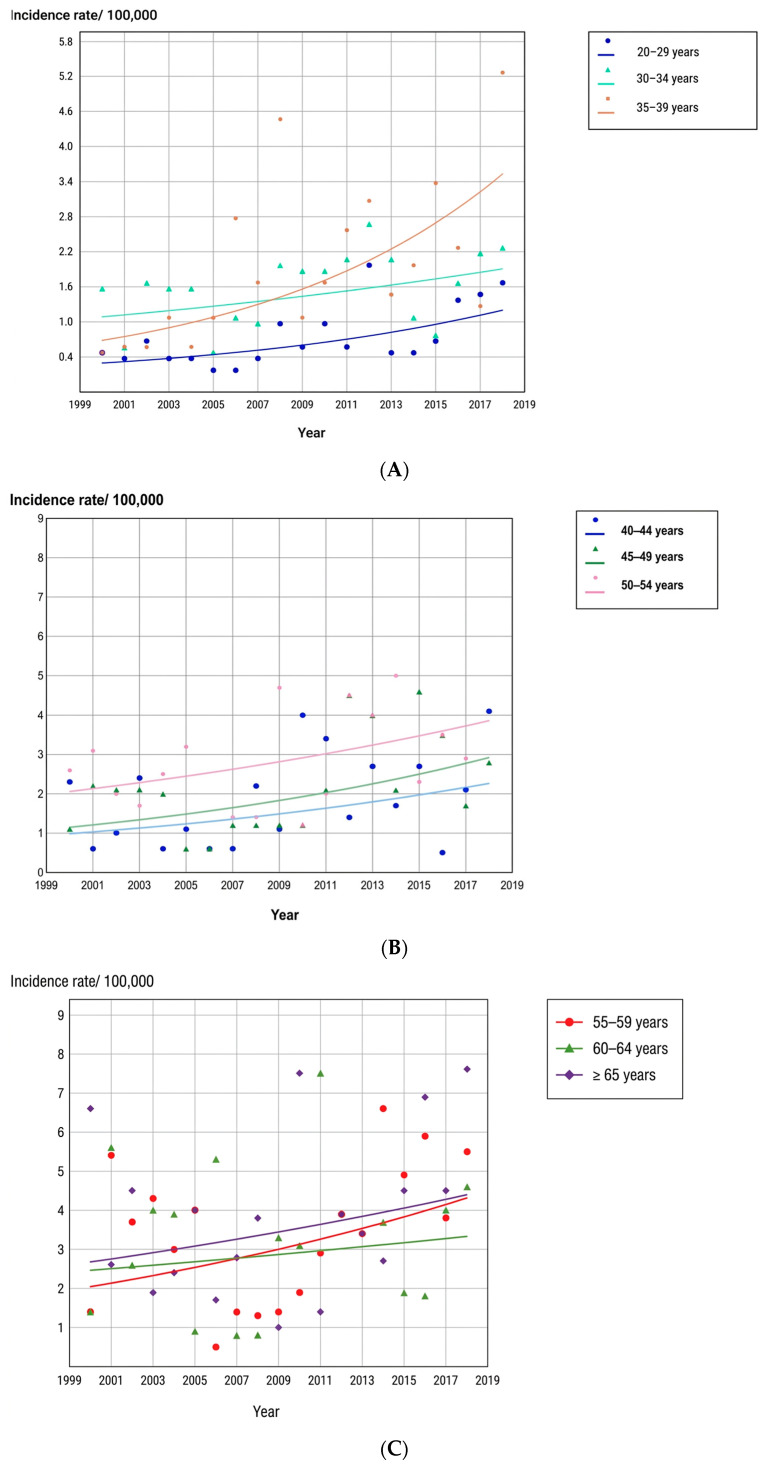
Trends in thyroid cancer incidence by male age group (**A**–**C**) in northern Tunisia, 2000–2018.

In males, significant upward trends were confined to the 20 to 29 and 35 to 39 age groups; all other male age groups showed stable incidence between 2000 and 2018. Table 4 presents age-group-specific data for males. Figure 4 displays these patterns graphically.

### 3.4. Trends in Tumor Extension by Sex

**Figure 5:**
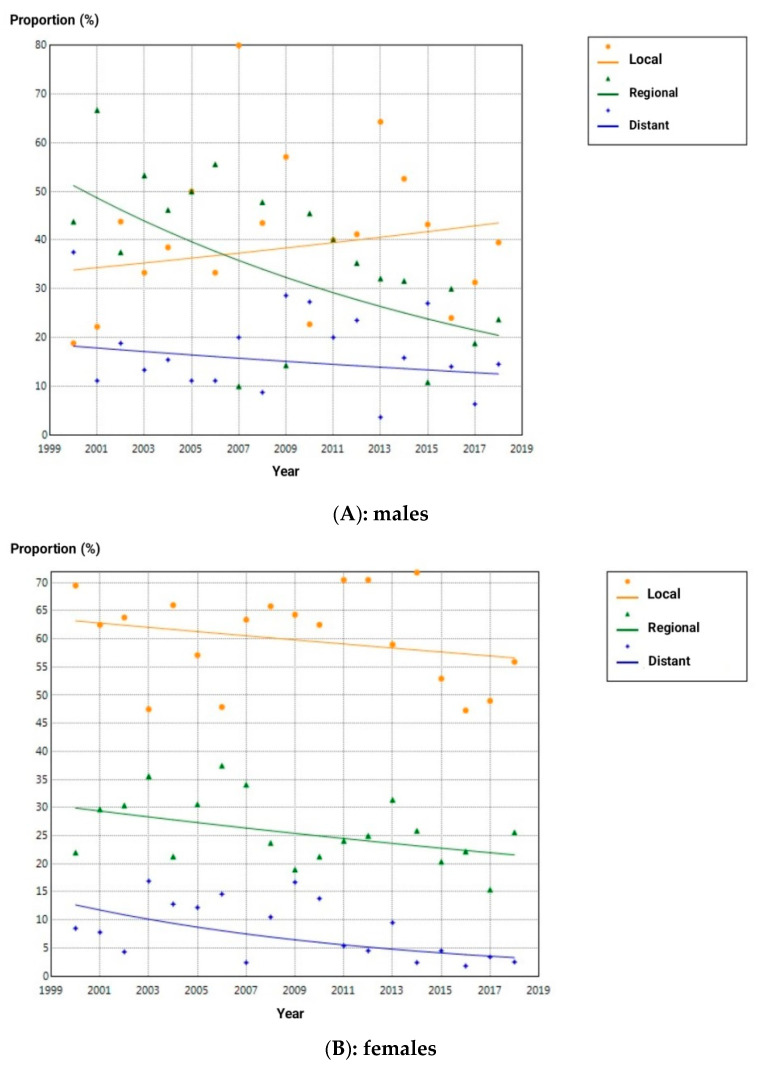
Trend in proportion of degree of thyroid cancer extension in males (**A**) and females (**B**) in northern Tunisia over the period 2000–2018.

Patterns of tumor extension evolved differently between the sexes. In males, the proportion of regionally advanced TC cases declined significantly from 53.8% in 2000 to 33.4% in 2018 (AAPC = −5.0%, *p* = 0.034). In females, the proportion of metastatic disease fell from 8.5% in 2000 to 4.4% in 2018 (AAPC = −7.2%, *p* = 0.033). Table 5 presents the full trend data for the extension by sex. Figure 5 displays the trajectories of extension proportion for males and females, respectively.

### 3.5. Incidence Trends by Histological Subtype

**Figure 6:**
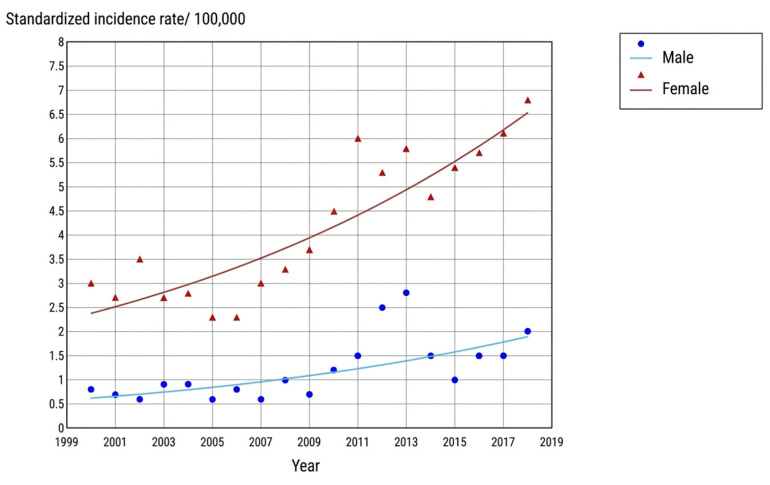
Trend in the incidence of papillary TC by gender in northern Tunisia during the period 2000–2018.

**Figure 7:**
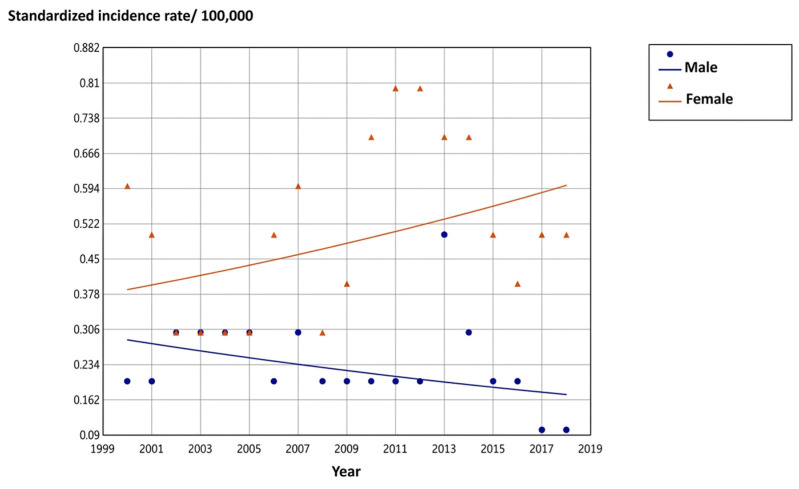
Trend in the incidence of follicular TC by gender in northern Tunisia during the period 2000–2018.

PTC incidence rose significantly in both sexes. The AAPC for PTC was 6.4% in males and 5.8% in females (both *p* < 0.001). FTC incidence showed no statistically significant differences between the sexes over 2000–2018. Table 6 presents ASIR and AAPC data by histological type and sex. Figure 6 and Figure 7 display PTC and FTC incidence trajectories for males and females, respectively.

### 3.6. Projections to 2040

Without any public health intervention, the BAPC model projects that the ASIR of TC will reach 7.6 (95% CI: 6.2–9.0) per 100,000 in males and 22.2 (95% CI: 15.5–31.7) per 100,000 in females by 2040. Table 7 presents projected ASIRs by sex through 2040.

## 4. Discussion

This population-based registry study documents a sustained, statistically significant rise in TC incidence across northern Tunisia between 2000 and 2018, driven predominantly by PTC in both sexes and concentrated among women of reproductive and peri-menopausal age. Three converging epidemiological signals characterize these findings: rising overall and sex-specific incidence, a histological pattern dominated by PTC growth while FTC remains unchanged, and a concurrent decline in the proportions of advanced-stage disease. Taken together, these signals could reproduce the overdiagnosis fingerprint documented across multiple international settings. A real increase in TC incidence is also very plausible since the increases in metabolic risk factors in Tunisia, such as obesity and lifestyle habits (diet, tobacco use, and physical inactivity).

### 4.1. Overall and Sex-Specific Incidence Trends

The ASIR of TC in northern Tunisia increased from 2.8 to 5.0 per 100,000 between 2000 and 2018, with an AAPC of 3.8% (*p* < 0.001). Female incidence rose from 3.7 to 7.8 per 100,000 (AAPC = 4.3%), and male incidence from 0.9 to 2.4 per 100,000 (AAPC = 3.0%). These represent sustained increases across nearly two decades that align with the global trajectory reported by Xu et al. [31], whose age–period–cohort analysis based on the Global Burden of Disease Study documented rising TC incidence across the vast majority of world regions from 1990 to 2019. The Tunisian female ASIR of 7.8 per 100,000 by 2018 is comparable to rates reported in Iran over a similar period [32] and approaches the levels observed in France and Switzerland in the same era [33,34]. The female predominance, with 78.5% of all cases occurring in women and a sex ratio of 0.3, is consistent with global patterns. In contrast to these rising trends, South Korea and the United States have reported recent declines in TC incidence following guideline revisions that discouraged aggressive evaluation of thyroid nodules [11,12]. Table 8 shows an explicit comparison with incidence patterns reported in other regions globally.

The observed gender disparity could be explained by many factors. According to the Tunisian Health Examination Survey (THES, 2016), the prevalence of obesity was twice as high in women as in men (34.6% vs. 17.6%), peaking in the 30–64 age group (42.6% in women and 22.2% in men) [16]. These findings support the major role of obesity in the upward trend in TC incidence, especially among women. However, other factors are in favor of the increase in incidence in both sexes, such as the high rates of a sedentary lifestyle (57.7%) and tobacco use (25.1%) [16]. Further studies are needed to investigate factors associated with sex disparity, especially differences in healthcare utilization.

### 4.2. Age- And Sex-Specific Incidence Patterns

The concentration of significant incidence increases among Tunisian women aged 30 to 65 years, with stability below 30 and above 65, delineates a reproductive-age and peri-menopausal vulnerability window. The 30 to 65 age range in Tunisian women corresponds precisely to the segment with the highest documented obesity prevalence (42.6%), and the largest rise in BMI observed between 2005 and 2016 [16]. High BMI has been independently associated with elevated TC incidence and more aggressive tumor phenotypes in multiple epidemiological analyses [14,15], providing a plausible biological mechanism for the age clustering observed here. Among males, significant increases were confined to the 20 to 29 and 35 to 39 age groups, with stability across all other brackets, a pattern that differs qualitatively from the female age distribution and may reflect distinct diagnostic pathways, including occupational screening and routine health check programs targeting young working-age men, rather than a uniform biological risk increase. Megwalu and Moon [35] documented comparable female age-group patterns in the United States, with peak increases in the 30 to 54 bracket preceding the ATA guideline-driven decline, reinforcing the interpretation that diagnostic intensity substantially shapes age-specific incidence distributions. These findings indicate that TC prevention messaging and nodule evaluation protocols in Tunisia should prioritize women in their fourth and fifth decades, where the convergence of modifiable metabolic risk factors and diagnostic detection opportunity is highest.

### 4.3. Histological Subtype Incidence Trends

PTC was associated with observed incidence increases in both sexes, with AAPCs of 6.4% in males and 5.8% in females (both *p* < 0.001). FTC showed no significant variation across the study period in either sex. This histological dichotomy serves as an epidemiological indicator of overdiagnosis in the TC surveillance literature. Miranda-Filho et al. [7], analyzing 25 country-level datasets, confirmed that PTC accounted for virtually the entire rise in global incidence, while FTC and other subtypes remained stable or declined, a pattern now reproduced in the Tunisian registry data. The differential detectability of these subtypes explains the divergence: PTC is typically small and well-demarcated, highly visible on high-resolution ultrasound, whereas FTC requires histopathological assessment of capsular and vascular invasion and is far less likely to be detected incidentally on imaging [9,10]. Whether the Tunisian PTC rise reflects genuine pathological increase or enhanced detection of subclinical disease cannot be definitively determined from incidence data alone. The concurrent decline in the proportions of regional and metastatic TC cases reported in Section 3.4, however, could strengthen the overdiagnosis interpretation.

### 4.4. Tumor Extension Trends

The proportion of regionally advanced TC in males fell from 53.8% in 2000 to 33.4% in 2018 (AAPC = −5.0%, *p* = 0.034). In females, metastatic disease declined from 8.5% to 4.4% over the same period (AAPC = −7.2%, *p* = 0.033). Declining proportions of regional and metastatic extensions of TC cases at diagnosis in the context of rising overall incidence constitute the classical epidemiological fingerprint of overdiagnosis: as imaging-detected early-stage tumors accumulate in the registry, they progressively dilute the proportion of cases presenting with advanced disease [8,9]. Vaccarella et al. [8] described this exact pattern across multiple high-income countries and argued that it was overdiagnosis rather than improved treatment efficacy alone. The Tunisian data could reproduce this signature in both sexes. Importantly, overdiagnosis carries downstream consequences beyond any survival consideration: unnecessary thyroidectomy, lifelong thyroid hormone replacement therapy, radioiodine exposure, and the psychological burden of a cancer diagnosis all impose costs on individuals and health systems that are disproportionate to any benefit derived from identifying tumors that would never have caused symptoms [36]. These findings indicate that Tunisia should implement prospective tracking of stage distribution alongside overall incidence, and that clinical guidance on thyroid nodule evaluation should incorporate risk-stratified biopsy thresholds aligned with ATA recommendations to reduce unnecessary diagnostic escalation [12,13].

### 4.5. Projections and Future Burden

The BAPC model projects that, absent intervention, the ASIR will reach 7.6 (95% CI: 6.2–9.0) per 100,000 in males and 22.2 (95% CI: 15.5–31.7) per 100,000 in females by 2040. The female projection would place Tunisia within the upper tier of globally documented TC burdens, approaching current rates in Turkey and substantially exceeding those of most European countries [3,34]. These projections are model-dependent and assume trajectory continuity; structural changes in diagnostic practice, national obesity trends, or screening policy could substantially modify the realized incidence. The wide confidence interval for the female projection (15.5 to 31.7) reflects compounded uncertainty across a 22-year extrapolation and should be interpreted as a planning scenario. Comparative projection analyses from France, Switzerland, Taiwan, and Saudi Arabia have consistently demonstrated that countries that fail to act on early overdiagnosis signals incur substantially higher diagnostic and treatment costs than those that implement timely guideline reform [33,34,37,38]. These findings indicate that the 2040 projections constitute a credible worst-case reference frame for health system capacity planning, physician training in risk-stratified nodule evaluation, and revision of Tunisia’s National Non-Communicable Disease Strategy.

### 4.6. Strengths and Limitations

To address concerns about potential underreporting in earlier years, we confirm that the NTCR maintained consistent population coverage and applied standardized active case-finding methods throughout the study period. The exhaustiveness of the data is supported by a high morphological verification (MV) rate (95.0% in males and 95.6% in females), which remained stable over time. Furthermore, the average of 1.1 notification sources per case reflects a consolidated network of data providers. These quality indicators, which adhere to IARC standards, suggest that the observed trends in thyroid cancer incidence are genuine epidemiological shifts rather than artifacts of improved registry coverage or data-collection methods.

Meanwhile, several methodological considerations bound the interpretation of these findings. First, the NTCR lacks a unique national citizen identifier, which complicates duplicate detection and prevents direct record linkage with the national death registry. The absence of mortality data within the registry framework limits the ability to formally confirm overdiagnosis through the gold-standard incidence-mortality decoupling method. Second, the quality of hospital records limited complete TNM stage capture, necessitating the use of broad extension categories (localized, regional, metastatic) rather than full pathological staging, which precluded subgroup analyses by TNM classification. Third, the NTCR covers only northern Tunisia; the center and southern regions have distinct sociodemographic profiles and access-to-care environments, and extrapolating these findings to the national TC burden requires caution. Fourth, individual-level data on modifiable risk factors, including BMI, tobacco exposure, radiation history, iodine intake, and reproductive variables, were not available within the registry framework, preventing direct risk factor attribution for the observed trends. Fifth, the analytical dataset covers the period 2000 to 2018. This boundary reflects a methodologically justified constraint that deserves explicit acknowledgment: population-based cancer registries operating under IARC-mandated quality assurance procedures require multi-source cross-referencing, duplicate resolution, incompatibility correction, and completeness verification before any year of data is certified for analytical use [21,22,26]. This validation process introduces a systematic lag, well-recognized across major international registry networks, including SEER, EUROCARE, and GLOBOCAN, between data collection and analytical readiness. The 2018 cutoff represents the last year for which the NTCR had completed its full validation cycle at the time of this analysis, ensuring that every included case meets the completeness and accuracy standards required for valid Joinpoint trend estimation. Publishing unvalidated or partially complete registry years would introduce systematic bias into APCs modeling and distort trend estimates. Future analyses incorporating subsequently validated registry cycles will extend the surveillance horizon and enable detection of any post-2018 inflection in incidence trajectories, including any effect of potential guideline changes or shifts in diagnostic practice that may have occurred in more recent years.

## 5. Conclusions

This population-based registry study analyzed TC incidence trends over 19 years of surveillance in northern Tunisia, examining sex- and age-specific, histological, and tumor extension patterns in 3639 primary invasive cases from an IARC-compliant registry. TC incidence rose substantially in both sexes, with the burden concentrated disproportionately in women and particularly in those aged 30 to 65 years. The papillary subtype drove the incidence increase in both males and females, while follicular TC remained stable across the full study period, a histological divergence that has been attributed to an overdiagnosis signature documented across diverse international settings. The concurrent decline in the proportions of metastatic disease among females and regionally advanced disease among males in the current study reinforces this interpretation, reproducing the epidemiological fingerprint of a growing pool of early-stage, imaging-detected tumors diluting the relative burden of advanced disease. Further studies are recommended to support this hypothesis in Tunisia. Meanwhile, the hypothesis of a genuine increase in TC incidence is also plausible. In fact, metabolic risk factors prevalent in the Tunisian population, rising obesity rates, physical inactivity, and tobacco use, provide a plausible substrate for a genuine biological component of the observed rise and argue for sustained investment in primary prevention. For clinicians and radiologists, the evidence calls for systematic adoption of risk-stratified thyroid nodule evaluation protocols aligned with current ATA guidance to reduce unnecessary biopsy and diagnostic escalation. For public health authorities, extending registry infrastructure to enable mortality linkage and capture individual-level risk factors and commissioning dedicated overdiagnosis studies are the most actionable next steps. The quantitative foundation established by this study provides, for the first time, a longitudinal population-level portrait of TC in northern Tunisia that is both analytically rigorous and directly usable for evidence-based cancer control policy.

## Figures and Tables

**Table 1:** Number and percentage of thyroid cancer cases by gender in Northern Tunisia, 2000–2018.

	Number	Percentage
Male	781	21.5
Female	2858	78.5
Total	3639	100

**Table 2:** Trends in thyroid cancer incidence by gender in northern Tunisia, 2000–2018.

Gender	ASIR (per 100,000 Person-Years)		AAPC	CI at 95%	*p*
	2000	2018			
Male	0.9	2.4	3.0	[1.7; 5.3]	<0.001
Female	3.7	7.8	4.3	[3.0; 5.7]	<0.001
Total	2.8	5.0	3.8	[2.8; 5.5]	<0.001

ASIR: age-standardized incidence rate; AAPC: Average annual percentage change; CI: confidence interval; *p*: level of significance (5%).

**Table 3:** Trends in thyroid cancer incidence by age group among women in northern Tunisia, 2000–2018.

Age Group (Year)	SIR (per 100,000 Person-Years)		AAPC	CI at 95%	*p*
	2000	2018			
20–29	3.5	7.4	5.2	[2.5; 5.9]	<0.001
30–34	5.0	9.0	5.2	[1.7; 8.8]	0.005
35–39	6.0	10.3	6.5	[3.0; 10.1]	0.001
40–44	7.0	12.3	3.0	[0.6; 5.4]	0.019
45–49	6.7	15.7	8.5	[5.3; 11.8]	<0.001
50–54	7.0	14.1	6.1	[3.7; 8.7]	<0.001
55–59	9.5	14.0	6.1	[0.8; 12.0]	0.021
60–64	8.8	14.9	4.2	[0.6; 7.9]	0.024
≥65	9.4	13.0	1.8	[−0.7; 4.4]	0.155

SIR: specific incidence rate; AAPC: Average annual percentage change; CI: confidence interval; *p*: level of significance (5%).

**Table 4:** Trends in thyroid cancer incidence by age group among males in northern Tunisia during 2000–2018.

Age Group (Year)	SIR (per 100,000 Person-Years)		AAPC	CI at 95%	*p*
	2000	2018			
20–29	0.5	1.7	7.7	[1.9; 13.7]	0.007
30–34	1.6	2.3	3.1	[−0.9; 7.3]	0.122
35–39	0.6	3.1	7.4	[2.1; 13.4]	<0.001
40–44	2.3	4.1	4.7	[−3.2; 13.5]	0.200
45–49	1.1	2.8	5.3	[−1.5; 12.5]	0.124
50–54	2.6	5.0	3.5	[−1.7; 9.1]	0.177
55–59	2.0	6.1	3.5	[−0.9; 8.1]	0.111
60–64	3.2	5.2	1.4	[−3.1; 6.2]	0.544
≥65	7.0	8.2	2.3	[−2.9; 7.9]	0.355

SIR: specific incidence rate; AAPC: Average annual percentage change; CI: confidence interval; *p*: level of significance (5%).

**Table 5:** Trend in the proportion of Thyroid Cancer extension by gender in northern Tunisia during the period 2000–2018.

Gender	Extension	Proportion (%)		AAPC	CI at 95%	*p*
		2000	2018			
Male	Local	23.1	44.1	1.4	[−2.0; 4.9]	0.454
	Regional	53.8	33.4	−5.0	[−9.4; −0.4]	0.034
	Metastatic	23.1	22.5	−2.1	[−8.2; 4.6]	0.545
	Total	100.0	100.0			
Female	Local	69.5	63.7	0.6	[−1.9; 0.7]	0.341
	Regional	22.0	31.9	−0.8	[−4.2; 0.6]	0.133
	Metastatic	8.5	4.4	−7.2	[−13.5; −0.5]	0.033
	Total	100	100			

AAPC: Average annual percentage change; CI: confidence interval; *p*: level of significance (5%).

**Table 6:** Trend in incidence of Thyroid Cancer by histological type and gender during the period 2000–2018.

Histological Type	Gender	ASIR (per 100,000) Person-Years)		AAPC	CI at 95%	*p*
		2000	2018			
Papillary thyroid carcinoma	Male	0.8	2.0	6.4	[3.1; 9.9]	<0.001
	Female	3.0	6.8	5.8	[4.1; 7.5]	<0.001
Follicular thyroid carcinoma	Male	0.2	0.1	−2.7	[−5.7; 0.4]	0.075
	Female	0.6	0.5	2.5	[−0.5; 5.5]	0.096

ASIR: age-standardized incidence rate; AAPC: Average annual percentage change; CI: confidence interval; *p*: level of significance (5%).

**Table 7:** Projections of the incidence of TC according to sex.

Year	Male		Female	
	ASIR	CI at 95%	ASIR	CI at 95%
2025	5.0	[3.9; 6.1]	10.0	[7.5; 12.8]
2030	5.9	[4.6; 6.9]	12.8	[9.7; 17.0]
2035	6.6	[5.3; 7.9]	16.7	[12.2; 23.1]
2040	7.6	[6.2; 9.0]	22.2	[15.5; 31.7]

ASIR: age-standardized incidence rate; CI: confidence interval.

**Table 8:** Trend in TC incidence across different countries worldwide.

Country	Period	Male	AAPC (CI at 95%)	Female	AAPC (CI at 95%)
Northern Tunisia (our study)	2000	0.9	6% (3.7; 8.3%)	3.7	4.3% (3.7; 5.7%)
	2018	3.0		7.8	
France	1990	1.7	4.4% (3.9; 4.8%)	5.6	4.4% (4.1; 4.6%)
	2018	5.6		18.5	
Iran	2010	1.2	NA	4	NA
	2019	2.15		5.11	
Switzerland	1980	2.6	1.4% (0.6; 2.2%)	4.8	2.6% (2.0; 3.1%)
	2016	4.3		11.9	
Saudi Arabia	2001	1.5	NA	4.4	NA
	2013	2.2		8.1	
Taiwan	1995	1.31	7.3% (6.9; 7.8)	4.79	6.8% (5.8; 7.8)
	2019	8.07		22.7	
South Korea	2012	27.8	−11.8% (−17.4; −5.9)	122.1	−16.3% (−21.2; −11.1)
	2016	19.0		69.8	

AAPC: Average annual percentage change CI: Confidence interval at 95%; NA: not available.

## Data Availability

The datasets analyzed during this study are not publicly available in accordance with national legislation governing cancer registry confidentiality (Article 4, Decree No. 2008-846), but are available from the corresponding author upon reasonable request.

## Abbreviations

APCs	Annual Percentage Changes
AAPC	Average Annual Percentage Change
ASIR	Age-Standardized Incidence Rate
ATA	American Thyroid Association
BAPC	Bayesian autoregressive age–period–cohort
BMI	Body Mass Index
CI	Confidence Interval
CIR	Crude Incidence Rate
FTC	Follicular Thyroid Cancer
GLOBOCAN	Global Cancer Observatory
IARC	International Agency for Research on Cancer
ICD-O	International Classification of Diseases for Oncology
NTCR	Northern Tunisia Cancer Registry
PTC	Papillary Thyroid Cancer
SD	Standard Deviation
TC	Thyroid Cancer
WHO	World Health Organization
